# First-principles study on the high-pressure physical properties of orthocarbonate Ca_2_CO_4_

**DOI:** 10.1038/s41598-023-38604-w

**Published:** 2023-07-14

**Authors:** Zi-Jiang Liu, Tian Li, Xiao-Wei Sun, Cai-Rong Zhang, Zhong-Li Liu, Ting Song, Xiao-Dong Wen

**Affiliations:** 1 School of Mathematics and Physics, Lanzhou Jiaotong University, Lanzhou, 730070 China; 2 Department of Applied Physics, Lanzhou University of Technology, Lanzhou, 730050 China; 3 Technology Innovation Center of Materials and Devices at Extreme Environment, Harbin Institute of Technology, Harbin, 150001 China

**Keywords:** Geophysics, Mineralogy

## Abstract

Orthorhombic Ca_2_CO_4_ is a recently discovered orthocarbonate whose high-pressure physical properties are critical for understanding the deep carbon cycle. Here, we study the structure, elastic and seismic properties of Ca_2_CO_4_-*Pnma* at 20–140 GPa using first-principles calculations, and compare them with the results of CaCO_3_ polymorphs. The results show that the structural parameters of Ca_2_CO_4_-*Pnma* are in good agreement with the experimental results. It could be the potential host of carbon in the Earth's mantle subduction slab, and its low wave velocity and small anisotropy may be the reason why it cannot be detected in seismic observation. The thermodynamic properties of Ca_2_CO_4_-*Pnma* at high temperature and high pressure are obtained using the quasi-harmonic approximation method. This study is helpful in understanding the behavior of Ca-carbonate in the Earth’s lower mantle conditions.

## Introduction

As the most important carbonate, CaCO_3_ is transported to the deep mantle by subduction slab and plays a crucial role in the global long-term carbon cycle^1^. It is also a mineral that plays a key role in biomineralization^2^. However, CaCO_3_ undergoes a series of phase transitions under high temperature and high pressure, forming various structures and polymorphs. So far, the predicted structures are mainly calcite, aragonite, post-aragonite, and pyroxene-like^3–9^, and these structures and polymorphs have been experimentally verified^3,4,6,9–15^. Some studies also considered the reaction of calcium carbonate with MgO, SiO_2_, and MgSiO_3_^7,10,16,17^, while ignoring the reaction with CaO. Previously, Al-Shemali and Boldyrev^18^ mentioned the possible formation of calcium orthocarbonate Ca_2_CO_4_ in the CaCO_3_ + CaO system under high pressure. Recently, using AIRSS^19^ and USPEX^20^ crystal structure prediction methods, Sagatova et al.^21^ discovered a new structure of calcium orthocarbonate Ca_2_CO_4_ (space group *Pnma*) stable at 13–50 GPa and 2000 K, the carbon atoms in this phase are fourfold coordinated, and the structure is similar to high temperature and high pressure α'_H_-Ca_2_SiO_4_ phase^22^. Afterward, they found that Ca_2_CO_4_-*Pnma* was stable in the pressure and temperature range of 20–100 GPa and 1000–2000 K using the density functional theory within quasi-harmonic approximation^23^. Subsequently, Binck et al.^24^ verified the results of Sagatova et al.^23^ with single-crystal diffraction experiments. In addition, other alkaline earth orthocarbonates, Mg_2_CO_4_-*Pnma*^25^, Mg_2_CO_4_-*P*2_1_/*c*^25^, Sr_2_CO_4_-*Pnma*^26^, and Ba_2_CO_4_-*Pnma*^26^ have also been predicted, of which Sr_2_CO_4_-*Pnma*^27^ and Mg_2_CO_4_-*P*2_1_/*c*^28^ have been experimentally verified.

The elastic, seismic, and thermodynamic properties of Ca_2_CO_4_-*Pnma* under high pressure have not been investigated so far. Even the elastic constants of CaCO_3_ polymorphs were only the experimental results of calcite^29–32^ and aragonite^33,34^ at ambient conditions. Using the first-principles method, Belkofsi et al. calculated the elastic constants of three calcite polymorphs(calcite-III, calcite-IIIb, calcite-VI)^35^, and Huang et al. studied the elastic properties of aragonite, post-aragonite and *P*2_1_/*c*^36^. The thermal expansion coefficient^37–42^ and heat capacity^37,43–45^ of calcite and aragonite were measured at ambient conditions, where there was a large difference between the fitted thermal expansion coefficient.

In this work, the structural properties, elastic properties, and seismic properties of Ca_2_CO_4_-*Pnma* at 20–140 GPa are studied using the first-principles calculations based on density functional theory and are compared with the results of CaCO_3_ polymorphs. The thermodynamic properties of Ca_2_CO_4_-*Pnma* are obtained by quasi-harmonic approximation method.

## Methods

First-principles calculations are done with using the VASP package^46,47^ with projector-augmented wave^48^. The exchange–correlation interactions adopt the Perdew-Burke-Ernzerhof functional within the generalized gradient approximation^49^. The electronic configurations of the atoms are Ca: 3*s*^2^3*p*^6^4*s*^2^, C: 2*s*^2^2*p*^2^, O: 2*s*^2^2*p*^4^, respectively. The cutoff energy of the plane-wave basis is set to 900 eV. The *k*-point mesh generation and data processing are obtained by vaspkit program^50^. The *k*-points mesh of Ca_2_CO_4_-*Pnma*, calcite, aragonite, *P*2_1_/*c*-l, post-aragonite, *P*2_1_/*c*-h and *C*222_1_ are set to 5 × 7 × 4, 9 × 9 × 2, 7 × 4 × 6, 7 × 10 × 3, 8 × 7 × 8, 8 × 10 × 4, and 6 × 5 × 10 using the Monkhorst–Pack scheme^51^, respectively. The convergence criteria for energy and force are 1.0⨯10^–8^ eV and 0.02 eV/Å, respectively. Based on the optimized lattice structure, the stress–strain method is used to obtain the elastic stiffness tensor. In order to ensure the accuracy of the elastic constants of Ca_2_CO_4_-*Pnma*, the elastic constants of calcite and aragonite are calculated and compared with the available experimental results^32,33^. As shown in Table S1 (see Supplementary Material), the calculated results are in good agreement with the experimental results^32,33^. The thermodynamic properties are calculated using the quasi-harmonic approximation method^52^ of the PHONOPY program^53,54^, and the force constants are calculated using the density functional perturbation theory^55^. The supercells of aragonite and Ca_2_CO_4_-*Pnma* adopt 2 × 2 × 2 and 2 × 2 × 1 unit cells, respectively. The convergence tests of the phonon spectrum calculations of aragonite and Ca_2_CO_4_-*Pnma* are shown in Tables S2, S3, and Figs. S1–S8 (see Supplementary Material).

## Results and discussion

### Structural properties

**Figure 1:**
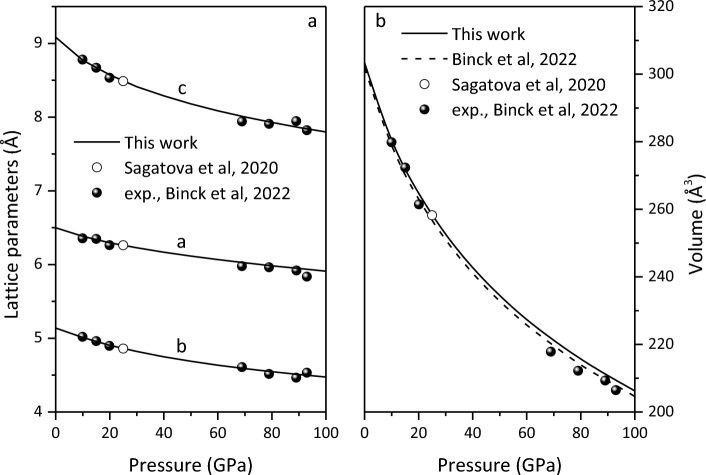
Lattice parameters (**a**) and equation of state (**b**) for Ca_2_CO_4_-*Pnma*.

The lattice parameters and equations of state for Ca_2_CO_4_-*Pnma* are presented in Fig. 1. It is found that the calculated results are in good agreement with the available experimental^24^ and previous theoretical results^21,24^, indicating the validity of the structure. The sensitivity of the axis to compression is c > b > a. The unit-cell volume at 0 GPa is 303.38 Å^3^ and the bulk modulus and its first pressure derivative are *K*_*0*_ = 113.40 GPa and *K*_*0*_^′^ = 4.00 by fitting the third-order Birch–Murnaghan equation, respectively, which are consistent with the results (*V*_0_ = 302.0(3) Å^3^, *K*_0_ = 108(1) GPa, and *K*_*0*_^′^ = 4.43(3)) of Binck et al.^24^.

In order to better understand the elastic and seismic properties of Ca_2_CO_4_-*Pnma*, the candidate CaCO_3_ structures (aragonite, *P*2_1_/*c*-l, post-aragonite, *P*2_1_/*c*-h, *C*222_1_, ‘− l = low pressure’, ‘− h = high pressure’) in the Earth's mantle are considered. The relative stabilities of the CaCO_3_ polymorphs considered in this work are evaluated from their enthalpies. According to Fig. S9 (see Supplementary Material), *P*2_1_/*c*-l stabilizes above 30 GPa and retains its stability up to 46 GPa, while *P*2_1_/*c*-h stabilizes above 75 GPa and retains its stability up to at least 140 GPa, which are consistent with the experimental and previous theoretical results^3,5^. CaCO_3_-*C*222_1_ above 137 GPa is stable relative to post-aragonite, but this does not make any sense^5,56^. Because in this interval, the modification *P*2_1_/*c*-h is more favorable. For comparison with calcium orthocarbonate, four modifications of CaCO_3_ must be considered, namely aragonite (20–35 GPa), *P*2_1_/c-l (35–45 GPa), post-aragonite (45–75 GPa) and *P*2_1_/c-h (75–140 GPa).

### Elastic properties

**Figure 2:**
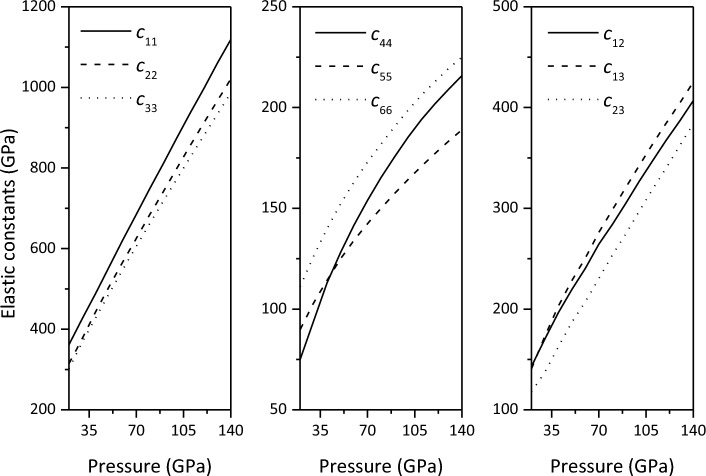
Elastic constants of Ca_2_CO_4_-*Pnma* at 20–140 GPa.

The calculated elastic constants of Ca_2_CO_4_-*Pnma* are shown in Fig. 2 and Table 1. Within the studied pressure range, $c_{11} > c_{22} > c_{33}$, indicating that compression is easier along the c-axis than along the a- and b-axes. These results are consistent with those of Fig. 1, where the lattice parameter c decreases faster than the lattice parameters a and b with increasing pressure. The calculated elastic constants of CaCO_3_ polymorphs are shown in Figs. S10–S13 and Tables S4–S7 (see Supplementary Material), respectively. Therefore, we believe that the calculated elastic constants are correct, but experimental verification is required.

**Table 1:** Calculated elastic constants (*c*_ij_ , in GPa), elastic modulus (*B* and *G*, in GPa), elastic anisotropy (*A*_100_, *A*_010_, and *A*_001_), and wave velocities(*V*_P_ and *V*_S_, in km/s), and seismic anisotropy (*A*_P_ and *A*_S_, in %) of Ca_2_CO_4_-*Pnma*.

	20 GPa	30 GPa	40 GPa	50 GPa	60 GPa	70 GPa	80 GPa	90 GPa	100 GPa	110 GPa	120 GPa	130 GPa	140 GPa
*c*_11_	361.283	426.524	490.296	556.045	621.896	684.963	749.298	810.915	874.479	936.647	996.922	1059.656	1118.588
*c*_12_	142.903	170.233	196.570	219.457	240.398	264.397	283.931	305.062	326.853	347.379	367.839	386.722	406.809
*c*_13_	140.540	173.079	203.134	228.336	250.733	276.075	298.478	320.722	342.530	364.008	384.721	404.475	425.289
*c*_22_	314.362	377.291	444.437	507.012	567.550	625.190	684.971	741.163	799.215	855.726	911.686	966.326	1020.541
*c*_23_	117.833	137.297	162.724	187.110	207.260	230.312	253.654	274.638	297.230	319.306	340.273	362.320	383.865
*c*_33_	301.281	368.295	429.494	489.713	548.568	605.510	662.449	717.991	772.260	826.578	880.161	933.167	985.928
*c*_44_	74.957	93.965	113.098	128.101	141.698	153.927	165.287	175.513	185.229	194.043	201.866	208.987	215.810
*c*_55_	89.751	103.061	114.329	124.943	134.165	142.370	150.016	157.360	164.243	170.868	177.235	183.270	188.939
*c*_66_	111.098	126.179	139.981	152.131	163.026	172.986	181.902	190.442	198.647	206.156	212.864	219.362	225.041
*B*	196.536	235.754	275.387	312.507	347.169	382.951	417.622	451.158	485.375	518.821	551.486	583.879	616.188
*G*	92.673	110.033	126.298	141.490	156.085	168.905	181.766	193.779	205.489	216.704	227.359	237.785	247.508
*A*_100_	0.786	0.8377	0.881	0.870	0.847	0.834	0.811	0.791	0.770	0.750	0.729	0.706	0.688
*A*_010_	0.945	0.8753	0.834	0.803	0.765	0.740	0.714	0.692	0.672	0.655	0.638	0.624	0.610
*A*_001_	1.1400	1.0893	1.034	0.975	0.920	0.886	0.840	0.809	0.779	0.751	0.726	0.701	0.679
*V*_P_	9.039	9.650	10.191	10.644	11.032	11.389	11.715	12.008	12.288	12.547	12.786	13.011	13.224
*V*_S_	4.863	5.176	5.436	5.656	5.849	6.002	6.148	6.275	6.392	6.499	6.595	6.684	6.763
*A*_P_	11.5	10.1	8.6	8.4	8.6	8.6	8.8	9.2	9.6	10.0	10.5	11.0	11.4
*A*_S_	19.61	14.71	10.65	10.41	12.44	13.77	15.83	17.28	18.81	20.29	21.67	23.18	24.41

**Figure 3:**
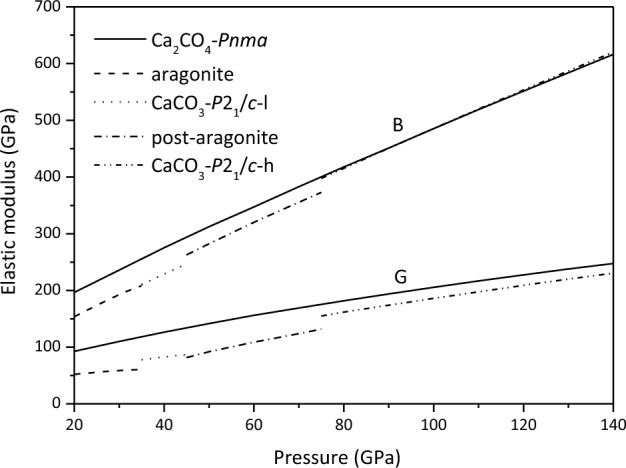
Bulk modulus *B* and shear modulus *G* of Ca_2_CO_4_-*Pnma* and CaCO_3_ polymorphs.

The bulk modulus (*B*) and shear modulus (*G*) of Ca_2_CO_4_-*Pnma* can be obtained by the Voigt^57^-Reuss^58^-Hill^59^ scheme. As can be seen from Fig. 3 and Table 1, *B* is greater than *G*, indicating that with the change of volume, Ca_2_CO_4_-*Pnma* is more and more difficult to be compressed, and *G* is the main factor for the deformation of Ca_2_CO_4_-*Pnma*. The *B* and *G* of Ca_2_CO_4_-*Pnma* at < 75 GPa are larger than those of CaCO_3_ polymorphs. The *B* of Ca_2_CO_4_-*Pnma* at 75–140 GPa is equal to that of *P*2_1_/*c-h*, and the *G* is slightly larger and almost parallel.

In order to evaluate the elastic anisotropy of Ca_2_CO_4_-*Pnma*, we adopt the scheme of Ravindran et al.^60^. The shear anisotropic factors of *A*_100_ in (100) plane, *A*_010_ in (010) plane, and *A*_001_ in (001) plane can be obtained from the following expression:

1 $$A_{100} = \frac{{4c_{44} }}{{c_{11} + c_{33} - 2c_{13} }}$$

2 $$A_{010} = \frac{{4c_{55} }}{{c_{22} + c_{33} - 2c_{23} }}$$

3 $$A_{001} = \frac{{4c_{66} }}{{c_{11} + c_{22} - 2c_{12} }}$$

**Figure 4:**
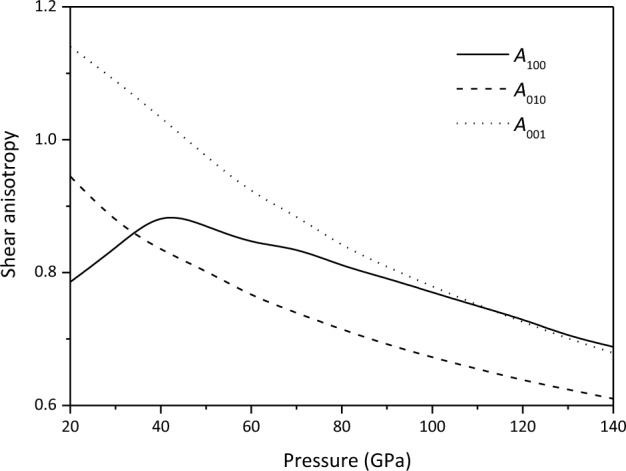
Shear anisotropic factors of Ca_2_CO_4_-*Pnma*.

The variation of shear anisotropic factors *A*_100_, *A*_010_ and *A*_001_ of Ca_2_CO_4_-*Pnma* with pressure is displayed in Fig. 4 and Table 1. *A*_010_ and *A*_001_ gradually decrease with increasing pressure, *A*_100_ first increases with the increase of pressure, and then gradually decreases at > 40 GPa. It can also be found that the elastic anisotropy of Ca_2_CO_4_-*Pnma* in the lower mantle conditions is very small, and the anisotropy of the (010) plane between [101] and [001] directions is the smallest.

The compressional and shear wave velocities of minerals can be calculated from the elastic constants and densities. The compressional (*V*_P_) and shear (*V*_S_) wave velocities of Ca_2_CO_4_-*Pnma* and CaCO_3_ polymorphs can be obtained from the Navier's equations^61^:

4 $$V_{P} = \sqrt {\frac{3B + 4G}{{3\rho }}} ,\quad V_{S} = \sqrt {\frac{G}{\rho }}$$

**Figure 5:**
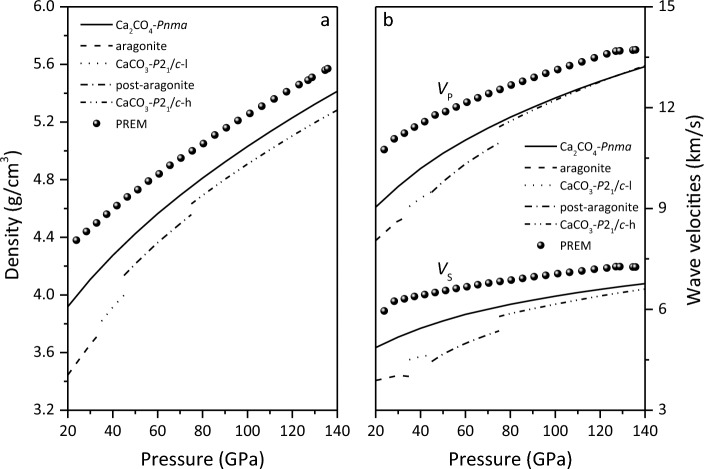
Densities (**a**) and wave velocities (**b**) of Ca_2_CO_4_-*Pnma*, CaCO_3_ polymorphs and PREM.

**Figure 6:**
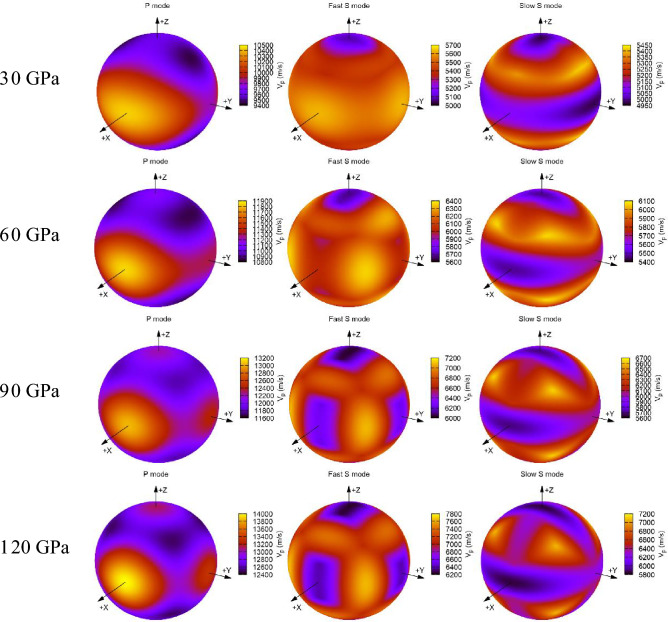
Wave velocities of Ca_2_CO_4_-*Pnma* along different crystallization directions at various pressures. Made using the AWESoMe program^64^.

The densities and wave velocities of Ca_2_CO_4_-*Pnma*, CaCO_3_ polymorphs and the Preliminary Reference Earth Model (PREM)^62^ are displayed in Fig. 5 and Table 1. From Fig. 5a, it is found that the densities of Ca_2_CO_4_-*Pnma* in the lower mantle is less than those of PREM, and greater than those of CaCO_3_ polymorphs. As shown in Fig. 5b, the *V*_P_ and *V*_S_ of CaCO_4_-*Pnma* and CaCO_3_ polymorphs are lower than those of PREM, and the *V*_P_ and *V*_S_ of Ca_2_CO_4_-*Pnma* are greater than those of *P*2_1_/*c-*l and post-aragonite, which are almost the same as those of *P*2_1_/*c-*h. The wave velocities in various crystallographic directions can be obtained by solving the Christoffel equation $\left| {C_{ijkl} n_{j} n_{l} - \rho V^{2} \delta_{ik} } \right| = 0$^63^. Figure 6 shows the wave velocities of Ca_2_CO_4_-*Pnma* along different crystallization directions at various pressures. The *V*_*P*_ of Ca_2_CO_4_-*Pnma* propagates the fastest in the [100] direction. The shear fast-wave velocity propagates the slowest in the [001] direction. With the increase of pressure, the propagation in the [100] and [010] directions become slower. The shear slow-wave velocity in [100] direction propagates more and more slowly as pressure increases.

The anisotropy *A*_*P*_ of the compressional waves and the polarization anisotropy *A*_*S*_ of the shear waves are defined as^65^:

5 $$A_{P} = \frac{{V_{P,\max } - V_{P,\min } }}{{V_{P,aggregate} }} \times 100\%$$

6 $$A_{S} = \frac{{\left| {V_{S1} - V_{S2} } \right|_{\max } }}{{V_{S,aggregate} }} \times 100\%$$

**Figure 7:**
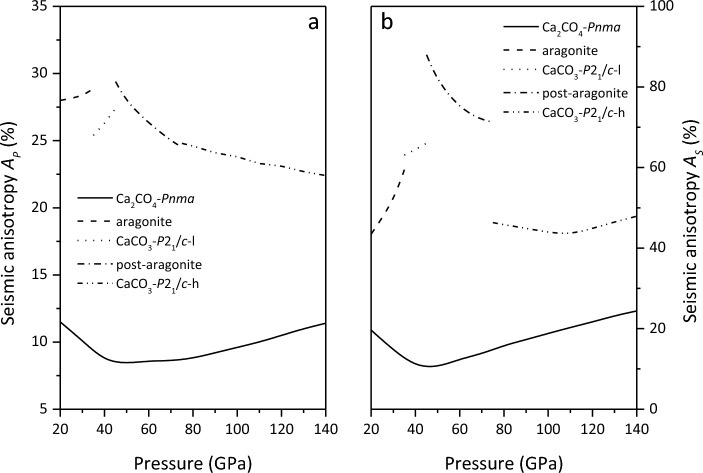
Seismic anisotropy *A*_*P*_ (**a**) and *A*_*S*_ (**b**) of Ca_2_CO_4_-*Pnma* and CaCO_3_ polymorphs.

Figure 7 and Table 1 show the *A*_*P*_ and *A*_*S*_ of Ca_2_CO_4_-*Pnma* and CaCO_3_ polymorphs. It can be seen that the seismic anisotropy *A*_*P*_ and *A*_*S*_ of Ca_2_CO_4_-*Pnma* are less than those of CaCO_3_ polymorphs, and decrease with the increase of pressure, and gradually increase at > 45 GPa. The nonlinear dependence of seismic anisotropy on pressure can be attributed to the nonlinear pressure sensitivity of the wave velocity, which is caused by the nonlinear pressure dependence of its elastic modulus, especially the shear modulus.

The seismic properties of Ca_2_CO_4_-*Pnma* indicate that it could be the potential host of carbon in the subduction slab and coexists with CaCO_3_ polymorphs, as suggested by Sagatova et al.^21,23^. It was also verified by Binck et al.^24^. The low wave velocity and small anisotropy of Ca_2_CO_4_-*Pnma* may be one of the reasons why it is impossible to detect the presence of carbonate in the lower mantle during the seismic observation of the subduction slab.

### Thermodynamic properties

**Figure 8:**
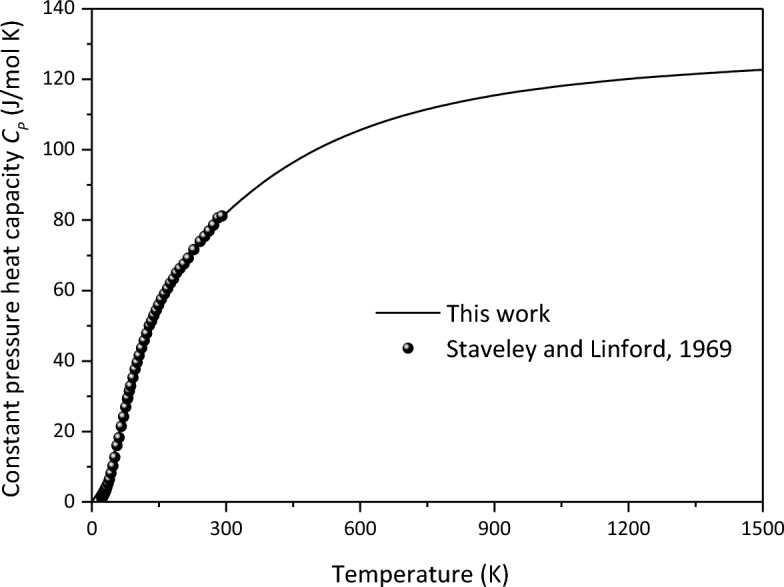
Constant pressure heat capacity *C*_*P*_ of aragonite at 0 GPa.

**Figure 9:**
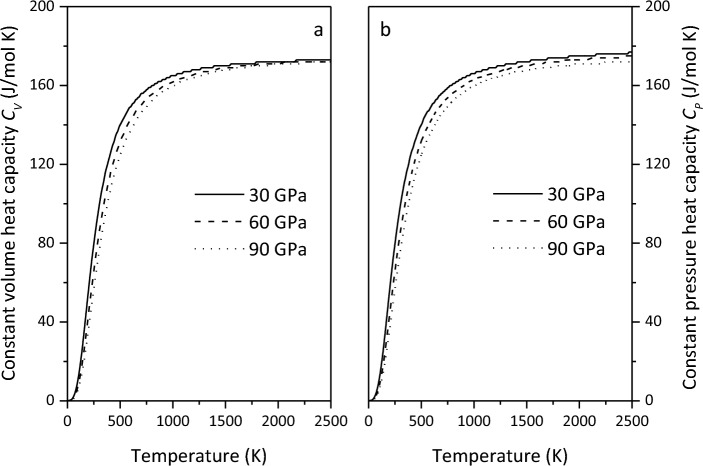
Constant volume heat capacity *C*_*V*_ (**a**) and constant pressure heat capacity *C*_*P*_ (**b**) of Ca_2_CO_4_-*Pnma* at various pressures.

**Figure 10:**
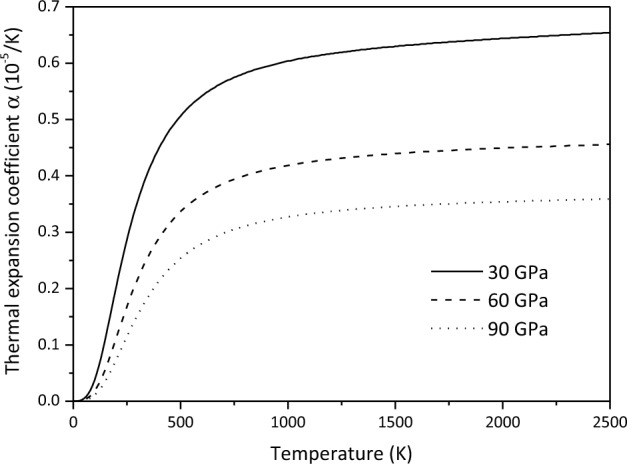
Thermal expansion coefficient *α *of Ca_2_CO_4_-*Pnma* at various pressures.

The thermodynamic parameters of minerals are a prerequisite for deriving the thermal state of the Earth's interior. In order to obtain the variation of thermodynamic parameters of Ca_2_CO_4_-*Pnma* with temperature and pressure, we first verify the constant pressure heat capacity *C*_*P*_ of aragonite at 0 GPa, and find that the calculated results are in good agreement with the experimental results^44^(Fig. 8). On this basis, the predicted heat capacity and thermal expansion coefficient $\alpha$ of Ca_2_CO_4_-*Pnma* are shown in Figs. 9 and 10, respectively.

Figure 9 shows that the constant capacity heat capacity *C*_*V*_ increases sharply with increasing temperature at low temperatures. Due to the suppression of non-harmonic effects under high pressure, the constant volume heat capacity *C*_*V*_ under high pressure and high temperature is very close to the Dulong Petit limit. The constant pressure heat capacity *C*_*P*_ is very close to the constant capacity heat capacity *C*_*V*_. In addition, the effects of temperature and pressure on constant capacity heat capacity *C*_*V*_ and constant pressure heat capacity *C*_*P*_ are opposite, and the impact of temperature is more noteworthy.

It can be seen from Fig. 10 that thermal expansion coefficient *α* at low temperature increases rapidly with the increase of temperature and tends to flatten rapidly with the increase of temperature. With the increase of pressure, the thermal expansion coefficient $\alpha$ decreases rapidly, and the influence of temperature becomes less and less obvious, resulting in linear high temperature behavior.

## Conclusions

On the basis of the determination of the stability for CaCO_3_ polymorphs in the lower mantle conditions and the verification of the structural parameters of Ca_2_CO_4_-*Pnma*, we study the elastic, seismic and thermodynamic properties of Ca_2_CO_4_-*Pnma*, and compared the results with those of CaCO_3_ polymorphs. The research shows that the densities of Ca_2_CO_4_-*Pnma* in the lower mantle are greater than those of CaCO_3_ polymorphs, and the seismic anisotropies are less than those of CaCO_3_ polymorphs. The wave velocities of Ca_2_CO_4_-*Pnma* and CaCO_3_ polymorphs are relatively low, and the wave velocities of Ca_2_CO_4_-*Pnma* and CaCO_3_-*P*2_1_/*c*-h are almost the same. This means that the presence of carbonate in the lower mantle is unlikely to be detected by seismic observations of subducted slab. By verifying the constant pressure heat capacity of aragonite at 0 GPa, the thermodynamic properties of Ca_2_CO_4_-*Pnma* at high temperature and high pressure are calculated using the quasi-harmonic approximation method. The results of this study are helpful to better understand the behavior of calcium carbonate in the lower mantle conditions.

## Supplementary Information

Supplementary Information.

## Data Availability

The datasets used and/or analyzed during the current study available from the corresponding author on reasonable request.
