# Supplementary material for: First-principles study on the high-pressure physical properties of orthocarbonate Ca2CO4
Source: Sci Rep. 2023 Jul 14;13:11422. doi: 10.1038/s41598-023-38604-w (PMC10349146; doi:10.1038/s41598-023-38604-w)
Supplement: Supplementary file 1 — Supplementary Information. [file 41598_2023_38604_MOESM1_ESM.docx]

Supplementary Materials: First-principles study on the high-pressure physical properties of orthocarbonate Ca_2_CO_4_

Zi-Jiang Liu^1^, Tian Li^1^, Xiao-Wei Sun^1^, Cai-Rong Zhang^2^, Zhong-Li Liu^3^, Ting Song^1^ & Xiao-Dong Wen^1^

^1^School of Mathematics and Physics, Lanzhou Jiaotong University, Lanzhou 730070, China

^2^Department of Applied Physics, Lanzhou University of Technology, Lanzhou 730050, China

^3^Technology Innovation Center of Materials and Devices at Extreme Environment, Harbin Institute of Technology, Harbin 150001, China

email: liuzj1024@hotmail.com

**Elastic constants of calcite and aragonite**

**Table S1**. Elastic constants(GPa), bulk modulus(GPa) and shear modulus(GPa) of calcite and aragonite.

|  | calcite | | aragonite | |
| --- | --- | --- | --- | --- |
|  | This work | Exp.^1^ | This work | Exp.^2^ |
| *c*_11_ | 146.42 | 144 | 167.45 | 171.1 |
| *c*_22_ |  |  | 110.85 | 110.1 |
| *c*_33_ | 82.11 | 84 | 98.53 | 98.4 |
| *c*_44_ | 33.38 | 33.5 | 36.29 | 39.3 |
| *c*_55_ |  |  | 19.70 | 24.2 |
| *c*_66_ |  |  | 38.05 | 40.2 |
| *c*_12_ | 53.42 | 53.9 | 57.94 | 60.3 |
| *c*_13_ | 51.49 | 51.1 | 30.86 | 27.8 |
| *c*_14_ | 17.02 | -20.5 |  |  |
| *c*_23_ |  |  | 44.00 | 41.9 |
| *B* | 73.33 | 73.3 | 69.49 | 68.9 |
| *G* | 33.51 | 32.0 | 33.20 | 35.8 |

**Convergence testing results of** **Ca_2_CO_4_-*Pnma* and aragonite in various supercells**

Figs. S1-S3 show the phonon dispersion curves and phonon density of states of Ca_2_CO_4_-*Pnma* in 1×1×1, 2×1×1 and 2×2×1 supercells at 20 GPa, respectively. Although there is no imaginary frequency in the phonon dispersion curve of Ca_2_CO_4_-*Pnma* among the three supercells, it is more reasonable to select a 2×2×1 supercell by observing the phonon state density. The convergence test results of plane-wave cutoff energy, k-point, and sigma for Ca_2_CO_4_-*Pnma* are shown in Tables S2, S3, and Fig. S4 respectively. As shown in Table S2, when the plane-wave cutoff energy is 600 eV, the energy difference (dE) of the two adjacent iterations is less than 0.002 eV/atom. It can be seen from Table S3 that when the k-point is 5×7×4, the energy difference (dE) is small. The integration scheme adopts Gaussian smearing techniques. As shown in Fig. S4, the sigma value has almost no effect on energy, so the default value of 0.05 eV is selected for this work. For aragonite, the phonon spectra of 1×1×1, 2×1×1, 2×2×1 and 2×2×2 supercells are shown in Figs. 5-8 when the plane-wave cutoff energy, k-point mesh, and smearing parameter are 600 eV, 7×4×6, and 0.05 eV, respectively. Only the phonon dispersion curve of aragonite in the 2×2×2 supercell has no imaginary frequency. The previous research results also proved the rationality of parameter selection in the phonon spectrum calculation of Ca_2_CO_4_-*Pnma* and CaCO_3_^3-7^.


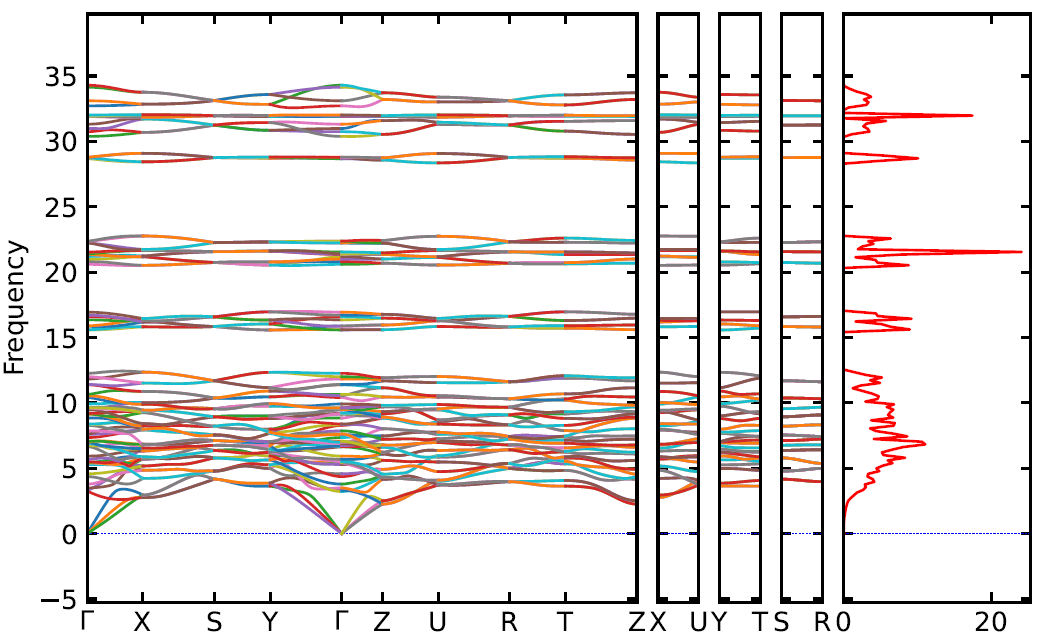


**Figure S1**. Phonon dispersion curves of Ca_2_CO_4_-*Pnma* in 1×1×1 supercell at 20 GPa.


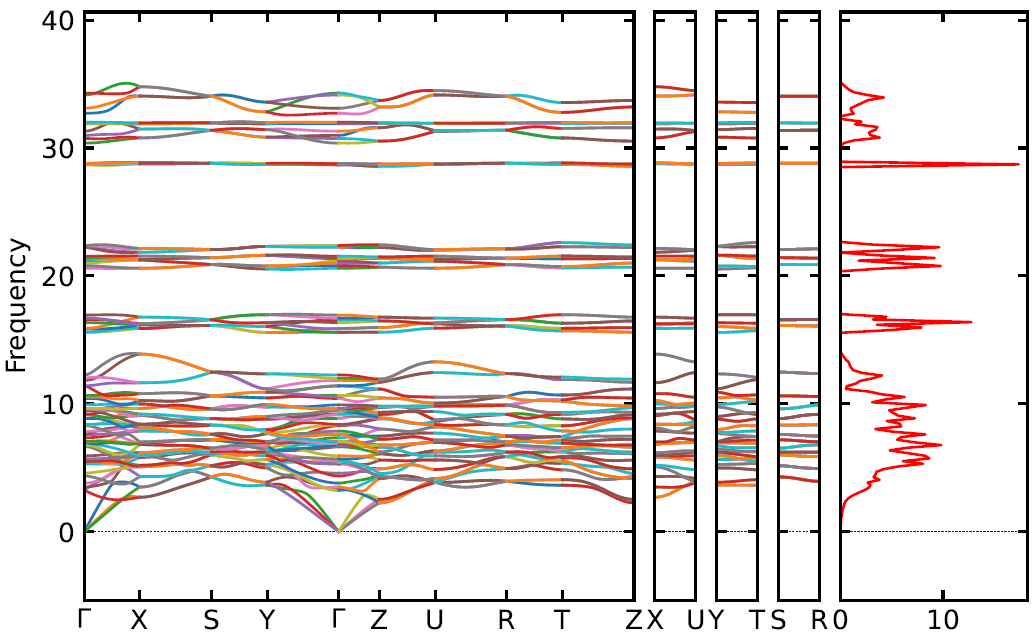


**Figure S2**. Phonon dispersion curves of Ca_2_CO_4_-*Pnma* in 2×1×1 supercell at 20 GPa.


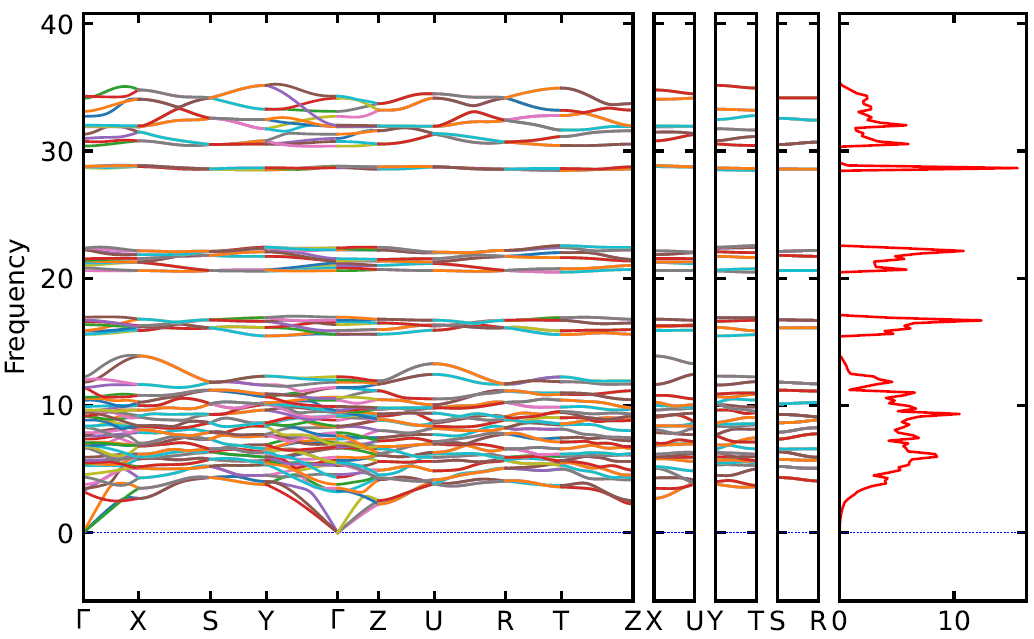


**Figure S3**. Phonon dispersion curves of Ca_2_CO_4_-*Pnma* in 2×2×1 supercell at 20 GPa.

**Table S2**. The calculated energy of Ca_2_CO_4_-*Pnma* at various plane-wave cutoff energy.

| Plane-wave cutoff (eV) | Energy (eV) | dE (eV) |
| --- | --- | --- |
| 450 | -781.10482158 |  |
| 500 | -780.80069564 | 0.0027154 |
| 550 | -780.87636335 | -0.0006756 |
| 600 | -781.09917386 | -0.0019894 |
| 650 | -781.31354113 | -0.0019140 |
| 700 | -781.48371724 | -0.0015194 |

**Table S3**. The calculated energy of Ca_2_CO_4_-*Pnma* at various k-points.

| k-points | Energy(eV) | dE (eV) |
| --- | --- | --- |
| 4×6×3 | -781.09949066 |  |
| 5×6×3 | -781.09936868 | 0.00012198 |
| 5×6×4 | -781.09917386 | 0.00019482 |
| 5×7×4 | -781.09898177 | 0.00019209 |
| 6×7×4 | -781.09945522 | -0.00047345 |
| 6×8×4 | -781.09913035 | 0.00032487 |

**Figure S4**. Sigma dependence of the energy of Ca_2_CO_4_-*Pnma* in 2×2×1 supercell at 20 GPa.


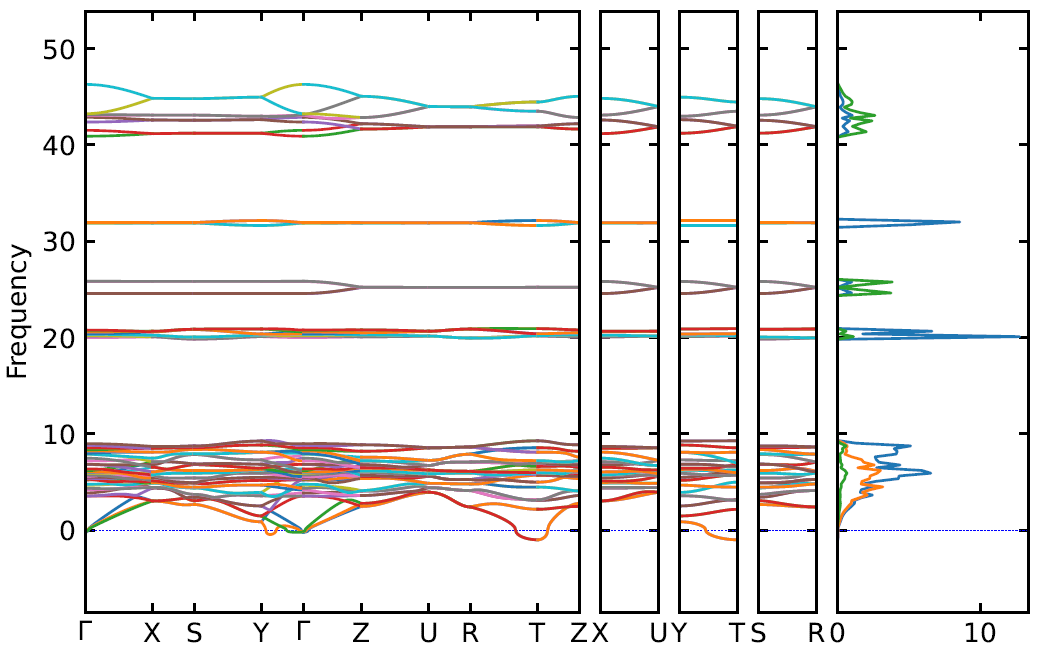


**Figure S5**. Phonon dispersion curves of aragonite in 1×1×1 supercell at 0 GPa.


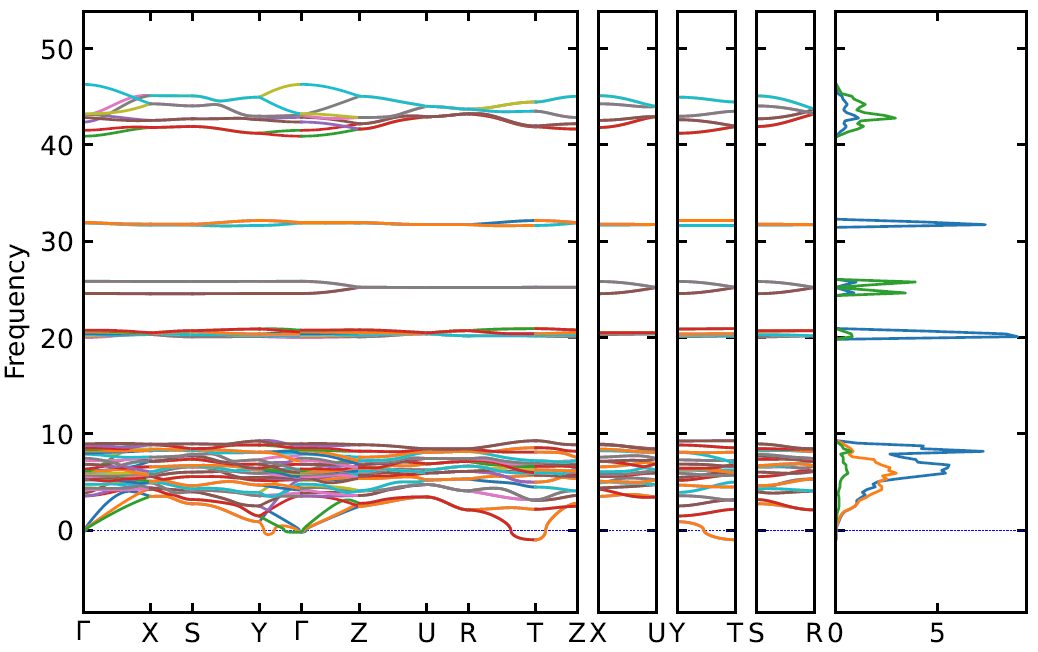


**Figure S6**. Phonon dispersion curves of aragonite in 2×1×1 supercell at 0 GPa.


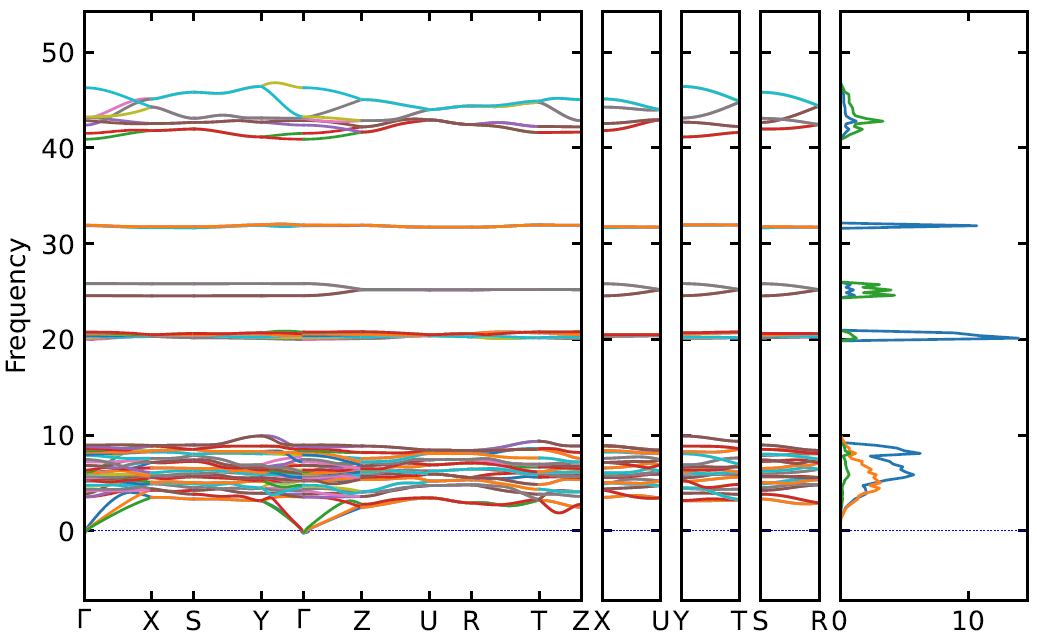


**Figure S7**. Phonon dispersion curves of aragonite in 2×2×1 supercell at 0 GPa.


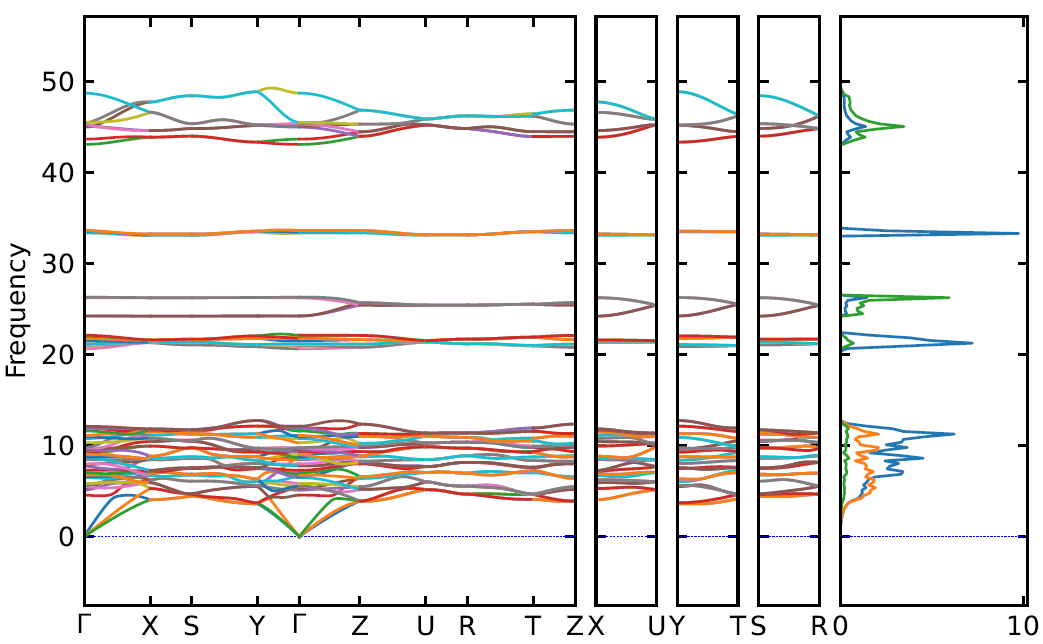


**Figure S8**. Phonon dispersion curves of aragonite in 2×2×2 supercell at 0 GPa.

**Enthalpy of CaCO_3_ polymorphs**

**Figure S9**. Enthalpy per formula unit of CaCO_3_ phases relative to post-aragonite.

**Elastic properties of CaCO_3_ polymorphs**

**Figure S10**. Elastic constants of aragonite at 20 to 35 GPa.

**Figure S11**. Elastic constants of CaCO_3_-*P*2_1_/*c*-l at 35 to 45 GPa.

**Figure S12**. Elastic constants of post-aragonite at 45 to 75 GPa.

**Figure S13**. Elastic constants of CaCO_3_-*P*2_1_/*c*-h at 75 to 140 GPa.

**Table S4**. Calculated elastic constants (*c*_ij_ , in GPa), elastic modulus(*B* and *G*, in GPa), wave velocities(*V*_P_ and *V*_S_, in km/s), and seismic anisotropy (*A*_P_ and *A*_S_, in %) of aragonite.

|  | 20 GPa | 30 GPa | 35 GPa |
| --- | --- | --- | --- |
| *c*_11_ | 314.286 | 385.969 | 412.082 |
| *c*_12_ | 139.818 | 173.686 | 206.509 |
| *c*_13_ | 85.744 | 114.282 | 117.762 |
| *c*_22_ | 206.743 | 253.478 | 278.559 |
| *c*_23_ | 133.965 | 179.990 | 200.924 |
| *c*_33_ | 180.743 | 219.041 | 232.379 |
| *c*_44_ | 60.568 | 69.775 | 74.026 |
| *c*_55_ | 38.301 | 45.213 | 47.923 |
| *c*_66_ | 70.563 | 91.038 | 102.039 |
| *B* | 153.687 | 193.851 | 208.366 |
| *G* | 51.944 | 59.489 | 60.514 |
| *V*_P_ | 8.044 | 8.643 | 8.764 |
| *V*_S_ | 3.883 | 4.033 | 4.005 |
| *A*_P_(%) | 28.0 | 28.3 | 28.9 |
| *A*_S_(%) | 43.47 | 51.63 | 59.47 |

**Table S5**. Calculated elastic constants (*c*_ij_ , in GPa), elastic modulus(*B* and *G*, in GPa), wave velocities(*V*_P_ and *V*_S_, in km/s), and seismic anisotropy (*A*_P_ and *A*_S_, in %) of CaCO_3_-*P*2_1_/*c*-l.

|  | 35 GPa | 40 GPa | 45 GPa |
| --- | --- | --- | --- |
| *c*_11_ | 381.108 | 420.017 | 456.709 |
| *c*_12_ | 193.162 | 206.114 | 219.900 |
| *c*_13_ | 102.808 | 112.696 | 122.600 |
| *c*_15_ | -1.600 | 1.911 | 4.375 |
| *c*_22_ | 296.504 | 322.597 | 349.136 |
| *c*_23_ | 199.417 | 220.233 | 240.935 |
| *c*_25_ | 6.908 | 6.848 | 6.760 |
| *c*_33_ | 275.147 | 291.682 | 307.096 |
| *c*_35_ | -21.019 | -20.420 | -20.461 |
| *c*_44_ | 110.234 | 121.562 | 132.572 |
| *c*_46_ | 3.074 | 3.828 | 4.762 |
| *c*_55_ | 63.068 | 61.715 | 58.917 |
| *c*_66_ | 119.233 | 127.795 | 136.757 |
| *B* | 210.345 | 228.823 | 246.736 |
| *G* | 77.716 | 82.673 | 86.947 |
| *V*_P_ | 9.060 | 9.306 | 9.501 |
| *V*_S_ | 4.504 | 4.595 | 4.639 |
| *A*_P_ | 25.4 | 26.3 | 27.4 |
| *A*_S_ | 63.11 | 64.47 | 66.03 |

**Table S6**. Calculated elastic constants (*c*_ij_ , in GPa), elastic modulus(*B* and *G*, in GPa), wave velocities(*V*_P_ and *V*_S_, in km/s), and seismic anisotropy (*A*_P_ and *A*_S_, in %) of post-aragonite.

|  | 45 GPa | 50 GPa | 60 GPa | 70 GPa | 75 GPa |
| --- | --- | --- | --- | --- | --- |
| *c*_11_ | 356.628 | 386.620 | 445.449 | 502.583 | 531.489 |
| *c*_12_ | 162.581 | 172.190 | 191.386 | 210.658 | 218.946 |
| *c*_13_ | 163.722 | 178.607 | 206.794 | 233.263 | 246.110 |
| *c*_22_ | 531.635 | 568.749 | 636.382 | 700.914 | 733.128 |
| *c*_23_ | 151.167 | 165.366 | 193.515 | 213.889 | 225.338 |
| *c*_33_ | 563.974 | 596.611 | 659.740 | 718.936 | 748.346 |
| *c*_44_ | 144.863 | 156.276 | 177.599 | 197.492 | 207.638 |
| *c*_55_ | 30.436 | 40.465 | 60.335 | 77.103 | 85.894 |
| *c*_66_ | 31.196 | 34.068 | 39.331 | 44.567 | 46.897 |
| *B* | 262.686 | 282.391 | 320.288 | 355.428 | 372.870 |
| *G* | 81.496 | 91.935 | 108.696 | 123.661 | 131.760 |
| *V*_P_ | 9.485 | 9.800 | 10.326 | 10.759 | 10.953 |
| *V*_S_ | 4.450 | 4.670 | 4.991 | 5.245 | 5.361 |
| *A*_P_ | 29.4 | 27.9 | 26.3 | 25.0 | 24.5 |
| *A*_S_ | 88.00 | 81.43 | 74.68 | 71.77 | 71.32 |

**Table S7**. Calculated elastic constants (*c*_ij_ , in GPa), elastic modulus(*B* and *G*, in GPa), wave velocities(*V*_P_ and *V*_S_, in km/s), and seismic anisotropy (*A*_P_ and *A*_S_, in %) of CaCO_3_-*P*2_1_/*c*-h.

|  | 75 GPa | 80 GPa | 90 GPa | 100 GPa | 110 GPa | 120 GPa | 130 GPa | 140 GPa |
| --- | --- | --- | --- | --- | --- | --- | --- | --- |
| *c*_11_ | 578.138 | 607.333 | 666.140 | 723.068 | 781.192 | 838.289 | 896.210 | 953.177 |
| *c*_12_ | 317.251 | 332.734 | 360.910 | 388.475 | 414.932 | 441.123 | 466.946 | 492.469 |
| *c*_13_ | 246.107 | 257.064 | 274.444 | 291.209 | 308.126 | 323.995 | 339.854 | 355.593 |
| *c*_15_ | 22.212 | 22.520 | 24.377 | 26.901 | 28.744 | 31.414 | 33.633 | 35.663 |
| *c*_22_ | 622.270 | 647.867 | 698.309 | 747.793 | 796.546 | 844.743 | 893.119 | 940.125 |
| *c*_23_ | 367.471 | 384.115 | 415.135 | 445.205 | 475.144 | 505.002 | 534.050 | 561.726 |
| *c*_25_ | 94.205 | 98.654 | 106.126 | 113.344 | 120.420 | 126.953 | 133.986 | 140.845 |
| *c*_33_ | 574.334 | 602.607 | 659.162 | 715.103 | 771.042 | 826.266 | 883.334 | 938.517 |
| *c*_35_ | 5.179 | 2.699 | -3.163 | -8.924 | -15.277 | -20.385 | -26.973 | -33.356 |
| *c*_44_ | 247.556 | 258.595 | 280.200 | 301.988 | 323.132 | 344.370 | 364.477 | 383.632 |
| *c*_46_ | 99.341 | 102.651 | 109.000 | 114.567 | 119.923 | 125.049 | 129.904 | 134.473 |
| *c*_55_ | 131.899 | 134.630 | 140.430 | 145.923 | 151.281 | 155.756 | 160.610 | 165.170 |
| *c*_66_ | 211.793 | 221.054 | 238.572 | 256.179 | 272.838 | 290.627 | 306.935 | 322.455 |
| *B* | 397.614 | 415.538 | 450.960 | 485.414 | 519.831 | 553.458 | 587.293 | 620.273 |
| *G* | 154.887 | 161.809 | 173.944 | 185.909 | 197.415 | 208.880 | 219.930 | 230.403 |
| *V*_P_ | 11.426 | 11.600 | 11.924 | 12.223 | 12.504 | 12.768 | 13.018 | 13.250 |
| *V*_S_ | 5.786 | 5.873 | 6.018 | 6.155 | 6.278 | 6.397 | 6.506 | 6.604 |
| *A_P_* | 24.8 | 24.6 | 24.1 | 23.8 | 23.3 | 23.1 | 22.7 | 22.4 |
| *A_S_* | 46.32 | 45.79 | 44.94 | 43.88 | 43.43 | 44.87 | 46.46 | 47.92 |

**References**

1. Hearmon, R. F. S., *The elastic constants of crystals and other anisotropic materials*. K. H. Hellwege, A. M. Hellwege, Eds., Landolt-Bornstein Tables,III/11 (Springer-Verlag, Berlin, 1979).

2. Liu, L.-g., Chen, C.-c., Lin, C.-C. & Yang, Y.-j. Elasticity of single-crystal aragonite by Brillouin spectroscopy. *Phys. Chem. Minerals* **32**, 97-102 (2005).

3. Yao, X., Xie, C., Dong, X., Oganov, A. R. & Zeng, Q. Novel high-pressure calcium carbonates. *Phys. Rev. B* **98**, 014108 (2018).

4. Sagatova, D., Shatskiy, A., Sagatov, N., Gavryushkin, P. N. & Litasov, K. D. Calcium orthocarbonate, Ca_2_CO_4_-*Pnma*: A potential host for subducting carbon in the transition zone and lower mantle. *Lithos* **370-371**, 105637 (2020).

5. Sagatova, D. N., Shatskiy, A. F., Gavryushkin, P. N., Sagatov, N. E. & Litasov, K. D. Stability of Ca_2_CO_4_-*Pnma* against the main mantle minerals from ab initio computations. *ACS Earth Space Chem.* **5**, 1709-1715 (2021).

6. Sagatova, D. N., Gavryushkin, P. N., Sagatov, N. E. & Banaev, M. V. High-pressure transformations of CaC_2_O_5_ - a full structural trend from double [CO_3_] triangles through the isolated group of [CO_4_] tetrahedra to framework and layered structures. *Phys. Chem. Chem. Phys.* **24**, 23578-23586 (2022).

7. König, J., Spahr, D., Bayarjargal, L., Gavryushkin, P. N., Sagatova, D., Sagatov, N., Milman, V., Liermann, H.-P. & Winkler, B. Novel calcium *sp*3 carbonate CaC_2_O_5_-*I*4̅2*d* may be a carbon host in earth’s lower mantle. *ACS Earth Space Chem.* **6**, 73-80 (2022).
